# Dynamic Changes in Volatiles, Soluble Sugars, and Fatty Acids in Glutinous Rice during Cooking

**DOI:** 10.3390/foods12081700

**Published:** 2023-04-19

**Authors:** Xianqiao Hu, Changyun Fang, Lin Lu, Zhanqiang Hu, Weixing Zhang, Mingxue Chen

**Affiliations:** Rice Product Quality Supervision and Inspection Center, Ministry of Agriculture and Rural Affairs, China National Rice Research Institute, Hangzhou 310006, China; hxhxqia@aliyun.com (X.H.);

**Keywords:** glutinous rice, water washing, presoaking, hydrothermal cooking, volatile profile, fatty acids, soluble sugars, flavor

## Abstract

Cooking is an important process before rice is consumed and constitutes the key process for rice flavor formation. In this paper, dynamic changes in aroma- and sweetness-related compounds were tracked during the entire cooking process (including washing with water, presoaking, and hydrothermal cooking). The volatiles, fatty acids, and soluble sugars in raw rice, washed rice, presoaked rice, and cooked rice were compared. After being washed with water, the total volatiles decreased while aldehydes and unsaturated fatty acids increased. Meanwhile, oligosaccharides decreased and monosaccharides increased. The changes in fatty acids and soluble sugars caused by the presoaking process were similar to those in the water-washing process. However, different changes were observed for volatiles, especially aldehydes and ketone. After hydrothermal cooking, furans, aldehydes, alcohols, and esters increased while hydrocarbons and aromatics decreased. Moreover, all fatty acids increased; among these, oleic acids and linoleic acid increased most. Unlike with washing and presoaking, all soluble sugars except fructose increased after hydrothermal cooking. Principal component analysis showed that cooked rice possessed a volatile profile that was quite different from that of uncooked rice, while washed rice and presoaked rice possessed similar volatile profiles. These results indicated that hydrothermal cooking is the pivotal process for rice flavor formation.

## 1. Introduction

Rice is the staple food of people in many countries. Greatly affecting palatability as well as consumers’ acceptability, flavor is one of the main characteristics of rice [1]. It consists of rice aroma and taste. Consumers prefer rice with a special, pleasant aroma and a sweet taste. Many works have reported on the characteristic aroma compounds and formation mechanism of rice aroma, the effects of genes, planting conditions, harvest conditions, and processing and storage conditions, etc., on the quality of rice aroma [2,3]. To date, nearly 500 volatiles have been reported in various rice samples, including aldehydes, furans, alcohols, esters, ketones, hydrocarbons, hetercocyclics, etc. [4]. Soluble sugars, such as sucrose and glucose, are considered the main compounds affecting the sweet taste of rice [5]. Moreover, they are reactants of the Maillard reaction during cooking, having an important impact on rice flavor.

Research on the volatile profile analysis of rice has largely focused on raw rice or cooked rice. The volatile profile of raw rice is quite different from that of cooked rice [6]. The content of 2-acetyl-1-pyrroline remarkably increased in cooked rice compared to raw rice. Ethyl butyrate, ethyl 3-methylbutanoate, 2-undecanone, 2-methylnaphthalene, 1-methylnaphthalene, ethyl benzoate, and ethyl benzeneacetate were characteristically detected in cooked rice, while high content of 2-ethyl-1-hexanol was uniquely found in raw rice. Zhao et al. found that the volatile profile of raw Chinese *japonica* rice was dominated by alkanes while that of cooked rice was predominated by aldehydes [7]. Rice is usually consumed in the form of cooked rice, processed after hydrothermal cooking. The processing technology affects the quality of the final aroma of cooked rice [8]. During cooking, the aroma formation of cooked rice is closely related to complex chemical reactions, including the Maillard reaction, lipid oxidation, and thermal degradation reactions, etc. [4]. The pre-cooking stage includes washing and soaking rice. Cooking methods include cooking in excess water and cooking using limited water absorption. The post-cooking stage includes treatments such as retorting, canning, cooling, freezing, drying, and storage of cooked rice [9]. Different processes not only affect the texture and nutritional value of final cooked rice [10,11] but also greatly impact the volatile profile [8,9].

Usually, raw rice is washed before cooking to remove impurities and any remaining hull or bran. Water washing significantly reduced volatiles for both head and broken rice [12]. A greater effect of water washing was observed for rice samples with a higher concentration of volatiles on the rice surface. This was the result of the effect of water washing on the total surface lipid of rice. About 60–70% of total surface lipids were removed by water washing [13]. Water washing is an effective process to improve aroma quality by reducing the end product’s off-flavor development. Presoaking rice in excess water before cooking is a traditional practice and is the most critical step affecting the quality of cooked rice. Presoaking for 30 min resulted in a significant increase in sewer/animal odor and summed negative odor, and a significant decrease in sweet taste and summed positive odor, mainly because of the increase in sulfur-containing free amino acids and their breakdown products [14]. However, Zhu et al. found that the volatiles of soaked rice were similar to those of unsoaked rice, and no new volatiles were produced [15]. They claimed that short-term soaking did not introduce significant unpleasant flavors. Hydrothermal cooking is applied during home cooking. Zeng et al. divided the entire hydrothermal cooking process into four stages, and the volatiles in the four stages were compared [16]. The major compounds identified at cooking stage I (25 min from the start of heating to the start of steam coming out of the rice cooker) were the low-boiling point volatiles such as saturated aliphatic aldehydes. Most saturated aliphatic aldehydes significantly decreased or even disappeared at cooking stage II (13 min from the start of steam coming out of the rice cooker to the end of steam coming out of the rice cooker), and decreased gradually at cooking stages III (10 min from the end of steam coming out of the rice cooker to the automatic stoppage of heating) and IV (keeping the rice warm for another 30 min from the automatic stoppage of heating). High-boiling point compounds such as fatty acids, significantly increased at cooking stage II and significantly decreased at cooking stage III, and then slightly increased at cooking stage IV. Key odorants such as (*E*)-2-nonenal, (*E,E*)-2,4-decadienal, 2-methoxy-4-vinylphenol, indole, and vanillin increased upon cooking. The water-to-rice ratio significantly affected rice texture but had little effect on flavor [17]. Cooking pressure had significant effects on the flavor of cooked rice [8]. High-pressure cooking significantly decreased off-flavor compounds compared to low-pressure cooking [18]. Cooling treatments also impact cooked rice quality. It was found that higher cooling rates prolonged rice flavor retention during storage [19]. A number of studies on the effect of cooking technologies on volatile profile have been reported. However, reports on the change in volatile profiles and the sweetness-related compounds of rice during cooking were limited.

Glutinous rice is one major type of cultivated rice, with longstanding cultural importance in Asia [16]. Glutinous rice has a distinctive aroma because of the high number of volatile compounds. Compared to *japonica* and *indica* rice, glutinous rice has a unique volatile profile, higher content of aldehydes and furans, and was more prone to deterioration during storage [20]. Fukuda et al. compared the volatiles in cooked rice with different amylose content [21]. Significant differences in volatile profile were found between glutinous and non-glutinous rice. Twenty-two volatiles were classified as glutinous-rich volatiles. Glutinous rice is the raw material of many important foods for annual events or festivities, e.g., rice wine, sweet fermented glutinous rice, rice cake, rice dumpling, Tang-yuan, and Yuan-xiao. According to its use, glutinous rice was usually consumed after being cooked or fermented. Fermentation plays an important role in influencing the flavor of fermented products. During fermentation, macromolecular polysaccharides, such as starch, gradually degrade into monosaccharides and disaccharides, mainly glucose and maltose [22]. Meanwhile, the main volatiles increase significantly. However, the change in aroma-related and sweetness-related compounds of glutinous rice during the cooking process was unclear.

In light of the above, in order to clarify the formation mechanism of glutinous rice flavor quality during cooking, in this paper, the aroma- and sweetness-related compounds in glutinous rice were tracked during the entire cooking process. The changes of volatiles, soluble sugars, and fatty acids in glutinous rice were analyzed after water washing, presoaking, and hydrothermal cooking.

## 2. Materials and Methods

### 2.1. Samples

Two glutinous rice samples (Chunjiangnuo and Fuxiangnuo) were used in this paper. The rice samples were husked and then milled before testing. The milled rice was defined as raw rice (RR). Part of the raw rice was washed with deionized water and dried with napkin (washed rice, WR). Part of the raw rice was washed, presoaked with deionized water for 30 min, and, finally, dried with napkin (presoaked rice, PR). Part of the raw rice was washed, presoaked with deionized water for 30 min, then cooked for 40 min, and simmered for 20 min (cooked rice, CR).

RP, WR, PR, and CR samples were ground into rice flour using a Cyclotec 1093 Sample Mill (Foss Tecator, Haganas, Sweden). Before grinding, the CR sample was freeze-dried. All rice flour samples were placed together to balance water content for 2 d, after which they were ready for subsequent analysis of volatiles, soluble sugar, and fatty acids.

Triglycerides were obtained from ANPEL Laboratory Technologies Inc. (Shanghai, China). Glucose, fructose, and sucrose were purchased from National Institute of metrology (Beijing, China). Raffinose was obtained from Dr. Ehrenstorfer GmbH (Augsburg, Germany).

### 2.2. Volatile Compounds Analysis

The volatile compounds analysis was performed for RP, WR, PR, and CR samples by using SPME-GC-MS method [20]. All rice samples were examined by using rice flour except CR sample, which was examined using fresh cooked rice. During volatile analysis of CR sample, 2.0 g milled rice kernels were weighed, placed in a 15 mL glass vial, washed, and then presoaked with 2.0 g deionized water for 30 min. After being sealed, the sample was cooked for 40 min and simmered for 20 min by placing on the steamer. Then, the fresh cooked rice was ready for volatile analysis. For RP, WR, and PR samples, 2.0 g rice flour were weighed and placed into a 15 mL glass vial; then, they were ready for the volatile analysis. The following extraction, GC-MS analysis, and identification of volatiles were the same as that reported before [20]. All experiments were performed in triplicate.

### 2.3. Soluble Sugars Analysis

The soluble sugars in RP, WR, PR and CR samples were examined by using ion chromatography-pulsed amperometric detector (IC-PAD) method [23]. Briefly, 0.5 g rice flour was weighed and placed into a 15 mL centrifugal tube, and 10 mL 50% ethanol was added. After being kept on a mechanical shaker for 30 min, the mixture was centrifuged at 1006× *g* for 10 min, and the supernatant was collected. The extraction was repeated twice, and supernatants collected were mixed evenly and filtered through a filter film (0.45 μm). The filtered supernatant was ready for the following IC-PAD. The filtered supernatant (10 μL) was injected and separated by using a Metrosep Carb 1 column (5.0 μm, 150 mm × 4.0 mm) with a mobile phase of 100 mmol/L NaOH at a flow rate of 1 mL/min. The detailed parameters for PAD were as follows: gold working electrode, Ag/AgCl reference electrode, E1 = 0.1 V, t1 = 0.4 s, E2 = 0.7 V, t2 = 0.2 s, E3 = −0.1 V, t3 = 0.4 s, tsample = 40 ms. All experiments were performed in triplicate.

### 2.4. Fatty Acids Analysis

The fatty acids in RP, WR, PR, and CR samples were examined by using gas chromatography-flame ionization detector (GC-FID) method [20]. Rice flour (1.0 g) was extracted with 5.0 mL toluene and 6.0 mL 10% acetyl chloride (in methanol solution) in sealed screw glass tube for 2 h at a temperature of 80 ± 0.5 °C. During this 2 h, the mixture was shaken well every half hour. After cooling to room temperature, the mixture, as well as 3 mL Na_2_CO_3_ solution (0.5 mol/L) which was used to clean the screw glass tube, were transferred to a 50 mL centrifuge tube. After being mixed evenly, the mixture was centrifuged at 2795× *g* for 5 min, and the supernatant was collected and filtered through a 0.45 μm filter film. Then, the filtered supernatant was ready for GC-FID examination. The filtered supernatant (1.0 μL) was injected and separated by using an HP-88 column (100 m × 0.25 mm × 0.2 μm) with ultrahigh-purity nitrogen gas (>99.999%) at a flow rate of 1.2 mL/min. The injection port temperature was 260 °C with a split ratio of 30:1. The detector temperature was 280 °C. The column temperature was programmed from 120 °C to 170 °C at 30 °C/min and maintained for 2 min, and increased to 200 °C at 6 °C/min and maintained for 2 min, then increased to 220 °C at 20 °C/min and further to 230 °C at 2 °C/min and kept for 5 min, then increased to 232 °C at 1 °C/min and maintained for 2 min, and, finally, increased to 240 °C at 3 °C/min and maintained for 5 min. All experiments were performed in triplicate.

### 2.5. Statistical Analysis

The mean value and the standard deviation of each volatile, soluble sugar, and fatty acid for RP, WR, PR, and CR samples were calculated. One-way analysis of variance was performed to test the differences among RP, WR, PR, and CR samples (*p* < 0.05).

## 3. Results and Discussion

Rice is one of the most important foods in the world. It is usually consumed as a staple food after cooking. In this paper, the changes in the volatiles, soluble sugars, and fatty acids of two glutinous rice samples were monitored during the entire cooking process. Usually, cooked rice is prepared by precooking, including being washed with water, presoaked, and then cooked hydrothermally [9]. Hence, the contents of volatiles, soluble sugars, and fatty acids were determined in raw rice, washed rice, presoaked rice, and cooked rice. In order to eliminate the error caused by water content, the rice flour samples were placed together to balance water content for 2 days before analysis. Meanwhile, the volatiles of cooked rice were investigated using fresh cooked rice.

**Figure 1:**
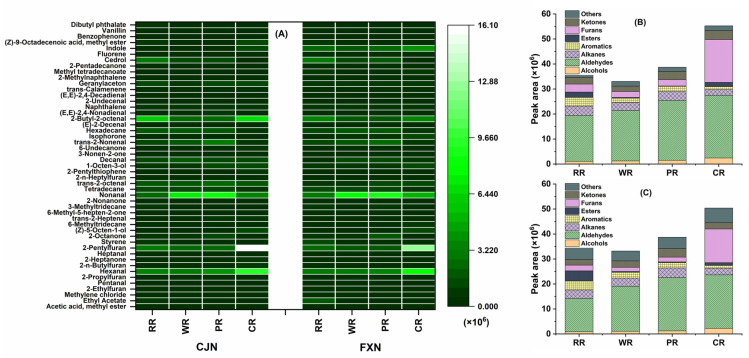
Change in volatile compounds during rice cooking process. (**A**) change in volatile profile of two rice samples, CJN and FXN; (**B**) change in chemical group profile of volatile in rice sample CJN; (**C**) change in chemical group profile of volatile in rice sample FXN. RR: raw rice; WR: washed rice; PR: presoaked rice; CR: cooked rice; CJN: Chunjiangnuo; FXN: Fuxiangnuo.

The volatiles in rice samples Chunjiangnuo and Fuxiangnuo were extracted using headspace SPME and analyzed by GC-MS. In total, 50 volatile compounds were identified by comparing their mass spectra and RI values with NIST (Figure 1A). More kinds of volatiles were identified in fresh cooked rice than in RR, WR, and PR samples. In total, 47 volatiles were identified in cooked Chunjiangnuo while 41, 39, and 38 were identified in RR, WR, and PR, respectively. Similarly, 47 volatiles were identified in cooked Fuxiangnuo while only 39, 38, and 37 were identified in RR, WR, and PR, respectively. As volatiles of CR samples were determined by using fresh cooked rice while those of RR, WR, and PR samples were determined by using rice flour, the matrix of samples varied greatly, causing a large difference of internal standard (IS) response between the CR sample and other samples. Hence, volatile content was not calculated by using IS. Peak areas of volatiles were analyzed in this paper. The total peak areas were (37.94 ± 4.37) × 10^6^ and (35.43 ± 3.74) × 10^6^ for RR samples Chunjiangnuo and Fuxiangnuo, respectively (Table S1). Aldehydes contributed most to the whole volatiles, accounting for 48.30% and 37.62% for Chunjiangnuo and Fuxiangnuo, respectively. This was consisted with the previous report that glutinous rice contained high aldehyde content [20].

### 3.1. Water Washing

Washing raw rice with water is often carried out before cooking to remove hull and other dust, and is thought to be an effective process to ameliorate the aroma of cooked rice [8]. After being washed, the total volatiles of rice decreased to (33.03 ± 0.85) × 10^6^ and (33.19 ± 3.37) × 10^6^ for Chunjiangnuo and Fuxiangnuo, respectively. Monsoor et al. also found that water washing significantly reduced the concentration of rice volatiles [12]. One important reason for this was the effect of water washing on the total surface lipid content. It was reported that about 60–70% of total surface lipids on the milled rice were removed by water washing [13]. Rice that was washed 3 times caused less flavor deterioration after the cooked rice was stored for 24 h than when the rice washed only once [24]. Water washing is perceived as a simple method to reduce off-flavor. Generally, a decrease in different kinds of volatiles, except aldehydes, was observed (Figure 1B,C). Total aldehydes increased from (18.33 ± 1.61) × 10^6^ and (13.33 ± 0.45) × 10^6^ to (20.16 ± 1.21) × 10^6^ and (18.03 ± 1.83) × 10^6^ for Chunjiangnuo and Fuxiangnuo, respectively (Table S1). Aldehydes have a pronounced fatty odor, and excessive concentrations lead to an off-flavor [25]. Hexanal, 2-butyl-2-octenal, and nonanal were the main aldehydes. A slight decrease was found after washing for hexanal. Hexanal is a linoleic acid oxidation product [26] and has a fruity odor at a low concentration. However, excessive content of hexanal leads to a significant, unpleasant odor of oil oxidation. A significant decrease in 2-butyl-2-octenal was found after water washing. It decreased from (6.25 ± 0.84) × 10^6^ and (3.53 ± 0.25) × 10^6^ to (2.30 ± 0.46) × 10^6^ and (1.81 ± 0.54) × 10^6^ for Chunjiangnuo and Fuxiangnuo, respectively. 2-Butyl-2-octenal is the condensation product of hexanal [27,28] and contributes odors of grass, citrus, and fruit. A greater effect of water washing was observed on the raw rice with higher volatiles. This might be due to the fact that there were more volatiles on the surface of raw rice. Interestingly, nonanal significantly increased after the water washing process.

**Figure 2:**
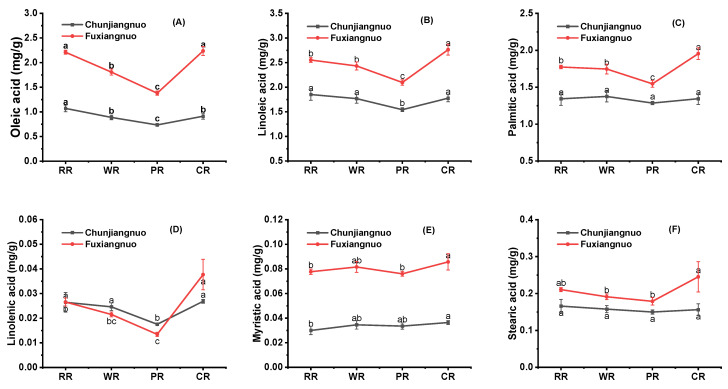
Changes in the fatty acids during rice cooking process. (**A**): oleic acid; (**B**): linoleic acid; (**C**): palmitic acid; (**D**): linolenic acid; (**E**): myristic acid; (**F**): stearic acid. RR: raw rice; WR: washed rice; PR: presoaked rice; CR: cooked rice. Means with the same letter were not significantly different (*p* < 0.05).

Linoleic acid, oleic acid and palmitic acid were the main fatty acids found in rice (Figure 2A–C). Myristic acid, stearic acid, and linolenic acid were also detected in rice (Figure 2D–F). Linoleic acid, oleic acid, and linolenic acid are unsaturated fatty acids while palmitic acid, myristic acid, and stearic acid are saturated fatty acids. It was found that unsaturated fatty acids decreased after water washing. This was consistent with the finding that water washing could reduce the total surface lipid and free fatty acid contents of milled rice [13]. However, palmitic acid and myristic acid slightly increased after water washing. Lipids were hydrolyzed by lipase to produce fatty acids and further decomposed into volatiles. Hence, the changes of fatty acids were not only affected by water washing but also by lipid oxidation. Washing rice with water is thought to shorten the soaking time required. During the water-washing period, enzymes were activated and released, and lipid oxidation and hydrolysis of starch and protein were accelerated [15]. The rice samples were not analyzed immediately after water washing. During the period from water washing to analysis, lipoxygenase catalyzed the peroxidation of polyunsaturated fatty acids containing a 1,4-pentadiene structure, such as linoleic and linolenic acids, into conjugated hydroperoxy fatty acids, which were further transformed into volatiles [29]. Higher activity of lipase resulted in more fatty acids. Meanwhile, higher activity of lipoxygenase resulted in more unsaturated fatty acids converted to hydroperoxy fatty acid. Under the comprehensive effect of all aspects, the unsaturated fatty acids in WR samples were lower than in RR samples. A greater decrease was obtained in oleic acid than in linoleic acid. This might be because of a greater degradation of oleic acid than of other fatty acids. Less degradation was obtained for saturated fatty acids than for unsaturated fatty acids. Unsaturated fatty acids are more easily enzymatically hydrolyzed due to the carbon–carbon double bond. Slightly higher contents of palmitic acid and myristic acid were observed in WR samples than in RR samples. The change of volatiles was the antagonism result of water washing and lipid oxidation. It was found that the oxidation products of oleic acid, such as decanal, nonanal, *trans*-2-nonenal, and heptanal, increased, while those of linoleic acid, such as hexanal, pentanal, 2-octenal, and 2-pentylfuran, decreased. No significant change was observed for the oxidation products of linolenic acid, such as *trans*-2-heptenal.

**Figure 3:**
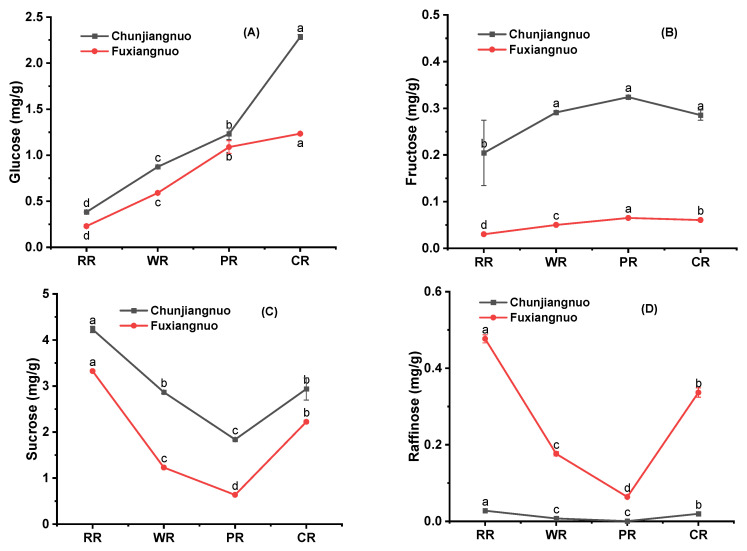
Changes in the soluble sugars during rice cooking process. (**A**): glucose; (**B**): fructose; (**C**): sucrose; (**D**): raffinose. RR: raw rice; WR: washed rice; PR: presoaked rice; CR: cooked rice. Means with the same letter were not significantly different (*p* < 0.05).

Soluble sugars were reported to have a significant influence on rice sweetness [30]. In addition, sugars are an important ingredient for the Maillard reaction. Glucose, fructose, sucrose, and raffinose are the main sugars in rice. The contents were 0.229–0.382 mg/g, 0.030–0.204 mg/g, 3.324–4.228 mg/g, and 0.028–0.477 mg/g for glucose, fructose, sucrose, and raffinose (Figure 3), respectively. The taste activity values (TAV) were determined by dividing the contents by the taste thresholds (0.9 mg/g, 1.1 mg/g, and 1.0 mg/g for fructose, glucose and sucrose, respectively) [31]. Results showed that sucrose (TAV > 1) was the main factor contributing to the characteristic sweetness of rice samples. During water washing, the soluble sugars on the rice surface were washed. Moreover, endogenous enzymes’ activities were enhanced [15], as there was a brief soaking time during water washing. Hydrolysis of starch and high molecular weight sugars were enhanced, resulting in a decrease in sucrose and increase in glucose [22]. In this study, it was found that monosaccharides (glucose and fructose) increased while oligosaccharide (sucrose and raffinose) decreased after water washing. Glucose and fructose increased to 0.590–0.874 mg/g and 0.05–0.291 mg/g, respectively. Sucrose and raffinose decreased to 1.230–2.865 mg/g and 0.008–0.176 mg/g, respectively. Sucrose was still the main factor contributing to the characteristic sweetness of rice samples.

During the water washing process, the volatiles, lipids, fatty acids, and soluble sugars on the rice surface were washed. Moreover, the activities of endogenous enzymes were enhanced during the brief soaking time. During the period between water washing and analysis, the starch, high molecular weight sugars, lipids, and fatty acids degraded under the action of the active enzymes. Degradation of starch and high molecular weight sugars resulted in a decrease in sucrose and raffinose and an increase in glucose and fructose. Under the comprehensive influence of water washing and degradation of lipids and fatty acids, unsaturated fatty acids decreased while palmitic acid and myristic acid increased. The total volatiles decreased while aldehydes increased to a certain extent. The increase in aldehydes was mainly due to the degradation of oleic acid. This resulted in an increase in its oxidation products, including decanal, nonanal, *trans*-2-nonenal, and heptanal.

### 3.2. Presoaking

Presoaking rice in water is a traditional practice before cooking. It is an important factor affecting cooking quality [32]. This process results in uniform cooking and less cooking time. During rice soaking, water diffuses into rice kernels due to the moisture gradient between the surface and the center of rice kernels. Meanwhile, sugars, volatiles, non-starch bound lipids and fatty acids leach from the rice grain [4]. Starch degradation, lipid oxidation, and decomposition of volatile precursors, i.e., hydroperoxides, are considerably promoted by enhancing the activity of the endogenous enzyme [4]. Similar to the effect caused by water washing, the change of volatiles, fatty acids, and soluble sugars was the comprehensive result of leaching out and degradation. After being presoaked for 30 min, volatiles and monosaccharides increased while oligosaccharides and fatty acids decreased. Total volatiles increased from (33.03 ± 0.85) × 10^6^ and (33.19 ± 3.37) × 10^6^ to (38.70 ± 3.68) × 10^6^ and (38.71 ± 4.69) × 10^6^ for Chunjiangnuo and Fuxiangnuo, respectively. Zhu et al. found a similar volatile profile between soaked and unsoaked brown rice [15]. They claimed that a certain degree of soaking did not introduce undesirable flavors. In their work, cooked rice with and without soaking were compared. The volatiles, induced by the subsequent Maillard reaction and thermal decomposition during hydrothermal cooking, provided cooked rice an intense flavor. This was different from that of WR and PR samples were compared in this paper. The increase in total volatiles was mainly caused by the increase in aldehydes and ketones. In addition, a slight increase in furans and alcohols also contributed to an increase in total volatiles. Among aldehydes, hexanal, *trans*-2-octenal, nonanal, and *trans*-2-nonenal increased more than other aldehydes. Hexanal and *trans*-2-octenal were decomposition products of linoleic acid, and nonanal and *trans*-2-nonenal were products of oleic acid. In addition to these aldehydes, 2-octanone, which contributes an odor of parmesan cheese [33], also increased significantly. Formation of ketones was also attributed to the oxidative degradation of unsaturated fatty acids [34]. All fatty acids were found to decrease. Among these, oleic acids and linoleic acid decreased most (Figure 2). This was ascribed to the activated lipoxygenase and oxidative properties of unsaturated acid and to the relative high content of oleic acids and linoleic acid. Compared to the water washing process, the presoaking process involved longer period of water soaking, resulting in more activated endogenous enzyme and, consequently, more degraded fatty acids. This might be the main reason for the decrease in fatty acids and increase in volatiles, especially in aldehydes and ketones. As in the change caused by water washing, glucose and fructose increased while sucrose and raffinose decreased after presoaking. This was the complex result of leaching into water and degradation of starch and polysaccharides. Glucose increased from (0.874 ± 0.003) mg/g and (0.590 ± 0.002) mg/g to (1.232 ± 0.063) mg/g and (1.088 ± 0.069) mg/g for Chunjiangnuo and Fuxiangnuo, respectively. Fructose increased from (0.291 ± 0.003) mg/g and (0.050 ± 0.001) mg/g to (0.324 ± 0.004) mg/g and (0.065 ± 0.002) mg/g for Chunjiangnuo and Fuxiangnuo, respectively. Monosaccharides increased because of greater amounts formed by starch and polysaccharide degradation than the amount leached out. Sucrose decreased from (2.865 ± 0.023) mg/g and (1.230 ± 0.020) mg/g to (1.839 ± 0.015) mg/g and (0.635 ± 0.006) mg/g for Chunjiangnuo and Fuxiangnuo, respectively. Raffinose decreased from (0.008 ± 0.003) mg/g and (0.176 ± 0.006) mg/g to (0.001 ± 0.001) mg/g and (0.064 ± 0.002) mg/g for Chunjiangnuo and Fuxiangnuo, respectively. Oligosaccharides decreased because of both leaching out and degradation. Sucrose still mainly contributed the characteristic sweetness of Chuangjingnuo, while no sugar played an important role in the sweetness of Fuxiangnuo. It was consistent with that presoaking for 30 min significantly decreased the sweet taste of rice in reported work [14].

On the whole, the changes of fatty acids and soluble sugars caused by the presoaking process were basically similar to those of the water washing process. However, the degree of change was much greater, because of an increased duration of water soaking. Fatty acids and oligosaccharides decreased and monosaccharides increased. Volatiles, especially aldehydes and ketone, increased. These were the degradation products of lipids.

### 3.3. Hydrothermal Cooking

Hydrothermal cooking of rice is mainly carried out by either absorption cooking (cooking by absorption with a predetermined amount of water) or excess water cooking (cooking in excess water at temperatures above the gelatinization temperature of the rice variety) [9]. In this paper, absorption cooking was used, and a water-to-rice ratio of 1 was used. Cooked rice samples contained much more volatiles than other samples (Figure 1 and Table S1). Hydrothermal cooking is thought to constitute the key process for the formation of rice flavor. During cooking, water gradually diffuses from the surface layer to the core of rice [4]. During boiling, water diffusion is promoted, the water distribution becomes even, and the rice kernels become homogeneous [35]. The starch undergoes high-temperature degradation and enzymatic hydrolysis during hydrothermal cooking. The reduced sugars generated undergo Maillard reaction with amino acids to produce volatiles such as furans, pyrazines, thiazoles, and pyrroles. It was found that total volatiles increased from (38.70 ± 3.68) × 10^6^ and (38.71 ± 4.69) × 10^6^ to (55.25 ± 12.24) × 10^6^ and (50.37 ± 10.44) × 10^6^ for Chunjiangnuo and Fuxiangnuo, respectively. The increase in volatiles was mainly caused by the increase in furans. Furans increased by 14.61 × 10^6^ and 11.60 × 10^6^ during hydrothermal cooking for Chunjiangnuo and Fuxiangnuo, respectively. The furan content in CR was 6.85- and 7.21-fold that in PR, and 5.40- and 5.98-fold that in RR. Furans were produced from both the Maillard reaction and lipid oxidation. 2-Pentylfuran, the most important alkylfuran identified in cooked rice, has a characteristic nut-like aroma in dilute concentrations and a less pleasant aroma, characteristic of soybeans, at higher concentrations. It can be obtained from the secondary oxidation products of linoleate hydroperoxides and Maillard reaction [4]. The breakdown temperature of the starch–lipid complex is lower than the cooking temperature [36]. Lipids leach out during cooking, due to the high temperature. Furthermore, the high temperature enhances the activity of lipase and lipoxygenase and accelerates the decomposition of hydroperoxides. All fatty acids were found to increase (Figure 2). Among these, oleic acids and linoleic acid increased most. The increase in fatty acids supplied more volatile precursors. The content of 2-pentylfuran was (16.10 ± 4.68) × 10^6^ and (12.80 ± 2.68) × 10^6^ for Chunjiangnuo and Fuxiangnuo, respectively, accounting for 94.1% and 95.2% of furans, respectively. During the final stage of hydrothermal cooking, the water in the system decreased after the temperature reached above 100 °C, which intensified the Maillard reaction in cooked rice [37]. Hence, more Maillard-related volatiles were formed in the final stage of cooking. This was consistent with the fact that 2-pentylfuran is found in the final stage of cooking [16]. In addition to 2-pentylfuran, hexanal and pentanal are also oxidation products of linoleic acid. They all increased during the hydrothermal cooking process. Hexanal increased from (4.28 ± 0.64) × 10^6^ and (3.58 ± 0.86) × 10^6^ to (9.32 ± 1.71) × 10^6^ and (7.91 ± 1.99) × 10^6^ for Chunjiangnuo and Fuxiangnuo, respectively. Pentanal increased from (0.07 ± 0.01) × 10^6^ and 0 to (0.18 ± 0.04) × 10^6^ and (0.16 ± 0.04) × 10^6^ for Chunjiangnuo and Fuxiangnuo, respectively. 2-Butyl-2-octenal was the second most abundant aldehyde in CR. It increased from (2.51 ± 0.49) × 10^6^ and (1.63 ± 0.07) × 10^6^ to (6.47 ± 0.65) × 10^6^ and (3.24 ± 0.45) × 10^6^ for Chunjiangnuo and Fuxiangnuo, respectively. 2-Butyl-2-octenal is believed to be a condensation product of hexanal. Hexanal may convert to 2-butyl-2-octenal during cooking. Nonanal, *trans*-2-nonenal, and decanal are oxidation products of oleic acid. Unlike the oxidation products of linoleic acid, they decreased after cooking. This might be due to the low oxidation of oleic acid compared to that of linoleic acid. Nonanal decreased from (8.11 ± 0.43) × 10^6^ and (8.83 ± 2.69) × 10^6^ to (3.45 ± 0.83) × 10^6^ and (4.89 ± 1.13) × 10^6^ for Chunjiangnuo and Fuxiangnuo, respectively. *Trans*-2-nonenal decreased from (2.69 ± 0.27) × 10^6^ and (2.62 ± 0.53) × 10^6^ to (0.55 ± 0.05) × 10^6^ and (0.68 ± 0.16) × 10^6^ for Chunjiangnuo and Fuxiangnuo, respectively. 2-Octanone, which has the odor of soap and gasoline, decreased from (1.34 ± 0.27) × 10^6^ and (1.85 ± 0.33) × 10^6^ to (0.19 ± 0.05) × 10^6^ and (0.22 ± 0.05) × 10^6^ for Chunjiangnuo and Fuxiangnuo, respectively. It was reported that methyl ketones were formed from fatty acids by enzymatic oxidative decarboxylation (β-oxidation) [38]. After hydrothermal cooking, furans, aldehydes, alcohols, and esters increased, and hydrocarbons and aromatics decreased, resulting in the characteristic odor of rice.

Unlike for washing and presoaking, all soluble sugars, except fructose, increased after hydrothermal cooking. During hydrothermal cooking, the starch undergoes high-temperature degradation and enzymatic hydrolysis, producing soluble sugars. Total soluble sugars increased from 3.423 mg/g to 5.820 mg/g for Chunjiangnuo, and from 1.869 mg/g to 4.006 mg/g for Fuxiangnuo. Meanwhile, reducing sugars, including glucose, fructose, and maltose, undergo Maillard reaction with amino acids to produce volatiles during cooking, decreasing the reducing sugars. The change of glucose, fructose, and maltose was the result of starch degradation and the Maillard reaction. TAV evaluation showed that sucrose and glucose (TAV > 1) were the main factors contributing the characteristic sweetness of cooked rice.

On the whole, under the action of the Maillard reaction and thermal degradation, volatiles varied greatly. Furans, aldehydes, alcohols, and esters increased while hydrocarbons and aromatics decreased. Hydrothermal cooking was the key process for the formation of rice flavor. Lipid oxidation was considerably promoted by the enhanced activity of lipase and lipoxygenase. All fatty acids increased. Among these, oleic acids and linoleic acid increased most. Unlike in washing and presoaking, all soluble sugars, except fructose, increased after cooking.

### 3.4. Principal Component Analysis

**Figure 4:**
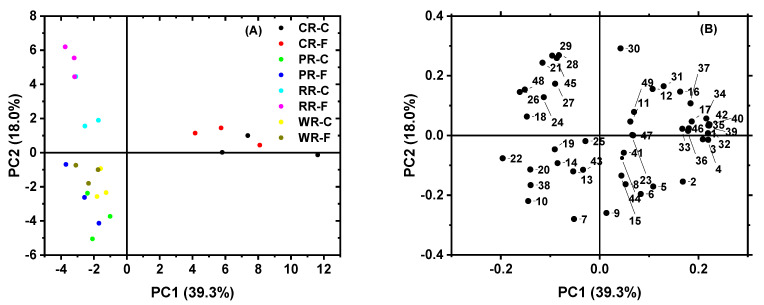
Principal component analysis of rice samples based on volatiles. (**A**): score plot; (**B**): loading plot. RR: raw rice; WR: washed rice; PR: presoaked rice; CR: cooked rice; C: Chunjiangnuo; F: Fuxiangnuo. Compounds **1**–**49**, see Table S1.

Principal component analysis (PCA) was conducted on the volatiles detected. As shown in Figure 4A, the first principal component (PC1) accounted for 39.3%, and the second principal component (PC2) accounted for 18.0%. According to PC1, cooked rice was distinct from uncooked rice (RR, WR, PR samples), indicating that the volatile profile of cooked rice was quite different from that of uncooked rice. As seen from Figure 4B, 2-pentylfuran, 6-methyl-5-hepten-2-one, 2-nonanone, 2-ethylfuran, 6-undecanone, hexanal, (*Z*)-5-octen-1-ol, 2-n-butylfuran, pentanal, 6-methyltridecane, 2-heptanone, 2-pentyl-thiophene, 2-n-heptylfuran, 1-octen-3-ol, 2-propylfuran, and vanillin greatly positively contributed to PC1 while naphthalene, benzophenone, 2-methylnaphthalene, and cedrol greatly negatively contributed to PC1. It was found that furans, ketone, aldehydes, and aromatics contributed most to PC1. Cooked rice contained more furans, aldehydes, and ketones, and uncooked rice contained more aromatics. This is inconsistent with the findings of Zhao et al. that the volatile profile of raw Chinese *japonica* rice was dominated by alkanes while that of cooked rice was dominated by aldehydes [7]. The difference might be the result of the use of different rice varieties. According PC2, the RR sample was distinct from the WR and PR samples. Methyl tetradecanoate, acetic acid methyl ester, ethyl acetate, methylene chloride, styrene, dbutyl phthalate, (*Z*)-9-otadecenoic acid methyl ester, 2-butyl-2-otenal, and edrol greatly positively contributed to PC2 while nonanal, decanal, *trans*-2-nonenal, *trans*-2-heptenal, heptanal, 2-octanone, *trans*-2-octenal, and 1-octen-3-ol greatly negatively contributed to PC2 (Figure 4B). Esters and aldehydes contributed most to PC2. The RR sample contained more esters, and the WR and PR samples contained more aldehydes. The WR and PR samples overlapped each other (Figure 4A), indicating that they possessed similar volatile profiles.

## 4. Conclusions

In this study, the formation mechanism of glutinous rice flavor quality during cooking was investigated. The volatiles, fatty acids, and soluble sugars in raw rice, washed rice, presoaked rice, and cooked rice were compared. After being washed with water, the total volatiles in rice decreased while aldehydes increased. Unsaturated fatty acids decreased and palmitic acid and myristic acid slightly increased. Oligosaccharides (sucrose and raffinose) decreased and monosaccharides (glucose and fructose) increased. During water washing, the lipids, fatty acids, and soluble sugars on the surface of the rice were washed out. Meanwhile, the activities of endogenous enzymes were enhanced during the brief soaking time. During the period between water washing and analysis, the starch, high molecular weight sugars, lipids, and fatty acids were degraded by the activated enzymes. The change of fatty acids and soluble sugars caused by the presoaking process was similar that of the water washing process. However, the degree of change was much greater during presoaking because of the increased duration of soaking. However, different changes were observed for volatiles. Total volatiles, aldehydes, and ketone increased. These are degradation products of lipids. The high temperature during hydrothermal cooking enhanced the activity of lipase and lipoxygenase and accelerated the decomposition of hydroperoxides. After hydrothermal cooking, furans, aldehydes, alcohols, and esters increased while hydrocarbons and aromatics decreased. Cooked rice possessed a volatile profile that was quite different from that of uncooked rice. Cooked rice contained more furans, aldehydes, and ketones, and uncooked rice contained more aromatics. Meanwhile, all fatty acids increased; among these, oleic acids and linoleic acid increased most. The starch underwent high-temperature degradation and enzymatic hydrolysis, producing soluble sugars. Unlike in washing and presoaking, all soluble sugars, except fructose, increased after cooking. It was found that hydrothermal cooking was the pivotal process for the formation of rice flavor. This study examined the changes of flavor-related compounds in rice during the entire cooking process. However, it is necessary to study the release mechanism of flavor by examining changes of matrix, such as starch structure, in the following work. This will provide theoretical support for the development of practical and scientific cooking technology in the future.

## Data Availability

Data is contained within the article.

## Supplementary Materials

The following supporting information can be downloaded at: https://www.mdpi.com/article/10.3390/foods12081700/s1, Table S1: Peak area of volatile compounds (×10^6^) in two rice samples during rice cooking process.
