# Supplementary material for: Dynamic Changes in Volatiles, Soluble Sugars, and Fatty Acids in Glutinous Rice during Cooking
Source: Foods. 2023 Apr 19;12(8):1700. doi: 10.3390/foods12081700 (PMC10137653; doi:10.3390/foods12081700)
Supplement: Supplementary file 1 [file foods-12-01700-s001.zip › foods-2327328-supplementary.pdf]

Supplementary Materials

Table S1 Peak area of volatile compounds (×10^6) in two rice samples during rice cooking process

No.	Compounds	Chunjiangnuo				Fuxiangnuo				RI_{cal}[a]	RI_{ref}[b]	Odor description[c]
		RR	WR	PR	CR	RR	WR	PR	CR			
1	(*Z*)-5-Octen-1-ol	0.25±0.01^{b}	0.28±0.02^{b}	0.36±0.03^{b}	1.08±0.28^{a}	0.22±0.02^{b}	0.24±0.01^{b}	0.27±0.09^{b}	1.12±0.31^{a}	1287	1287	fat, soap, lemon, green
2	1-Octen-3-ol	0.84±0.05^{b}	0.96±0.10^{b}	1.12±0.14^{ab}	1.32±0.30 ^{a}	0.65±0.01^{b}	0.81±0.03^{ab}	0.93±0.27^{ab}	1.10±0.31 ^{a}	1455	1455	mushroom
	Alcohols	1.09±0.06	1.24±0.11	1.47±0.17	2.40±0.57	0.87±0.01	1.05±0.04	1.20±0.35	2.22±0.61			
3	Pentanal	0.07±0.01^{b}	0.04±0.04^{b}	0.07±0.01^{b}	0.18±0.05^{a}	ND	0.05±0.01^{b}	ND	0.16±0.04^{a}	972	972	almond, malt, pungent
4	Hexanal	3.27±0.06^{b}	3.19±0.20^{b}	4.28±0.64^{b}	9.32±1.71^{a}	2.84±0.18^{b}	2.84±0.2 ^{b}	3.58±0.86^{b}	7.91±1.99^{a}	1078	1078	grass, tallow, fat
5	Heptanal	0.15±0.13^{b}	0.15±0.13^{b}	0.44±0.09^{a}	0.46±0.12^{a}	0.17±0.15^{b}	0.27±0.02^{ab}	0.51±0.20^{a}	0.44±0.11^{a}	1182	1182	fat, citrus, rancid
6	*trans*-2-Heptenal	0.49±0.02^{b}	0.52±0.01^{ab}	0.63±0.09^{a}	0.58±0.11^{ab}	0.31±0.03^{a}	0.35±0.02^{a}	0.48±0.20^{a}	0.45±0.11^{a}	1318	1318	green，soap, fat, almond

7	Nonanal	2.26±0.11^{c}	7.48±0.62^{a}	8.11±0.43^{a}	3.45±0.83^{b}	2.82±0.36^{c}	7.68±0.98^{ab}	8.83±2.69^{a}	4.89±1.13^{bc}	1391	1391	fat, citrus, green
8	*trans*-2-octenal	1.80±0.10^{a}	1.66±0.16^{a}	2.17±0.44^{a}	1.76±0.31^{a}	1.06±0.08^{a}	1.02±0.09^{a}	1.47±0.30^{a}	1.46±0.37^{a}	1425	1425	green, nut, fat
9	Decanal	1.20±0.22^{c}	1.64±0.01^{a}	1.53±0.03^{ab}	1.25±0.23^{bc}	0.42±0.37^{b}	1.31±0.22^{a}	1.37±0.36^{a}	1.06±0.23^{a}	1497	1497	soap, orange peel, tallow
10	*trans*-2-Nonenal	1.29±0.18^{c}	1.86±0.14^{b}	2.69±0.27^{a}	0.55±0.05^{d}	1.35±0.33^{bc}	1.91±0.46^{ab}	2.62±0.53^{a}	0.68±0.16^{c}	1531	1531	cucumber, fat, green
11	(*E*)-2-Decenal	0.49±0.17^{a}	0.42±0.14^{a}	0.17±0.30^{a}	0.30±0.05^{a}	0.20±0.17^{a}	ND	0.23±0.21^{a}	0.42±0.11^{a}	1640	1640	tallow
12	2-Butyl-2-octenal	6.25±0.84^{a}	2.30±0.46^{b}	2.51±0.49^{b}	6.47±0.65^{a}	3.53±0.25^{a}	1.81±0.54^{b}	1.63±0.07^{b}	3.24±0.45^{a}	1666	1666	green
13	(*E,E*)-2,4-Nonadienal	0.39±0.14^{a}	0.28±0.25^{a}	0.56±0.05^{a}	0.31±0.10^{a}	0.31±0.04^{a}	0.45±0.21^{a}	0.35±0.04^{a}	0.25±0.11^{a}	1696	1696	fat, wax, green
14	2-Undecenal	0.36±0.10^{ab}	0.25±0.22^{ab}	0.42±0.15^{a}	0.12±0.11^{b}	0.25±0.03^{a}	0.23±0.10^{a}	0.28±0.07^{a}	0.21±0.05^{a}	1749	1749	sweet
15	(*E,E*)-2,4-Decadienal	0.24±0.24^{a}	0.31±0.09^{a}	0.34±0.09^{a}	0.19±0.06^{a}	ND	0.06±0.11^{a}	ND	0.17±0.05^{a}	1805	1805	fried, wax, fat
16	Vanillin	0.08±0.04^{ab}	0.05±0.02^{b}	0.04±0.01^{b}	0.11±0.0 ^{a}	0.08±0.03^{ab}	0.05±0.02^{bc}	0.03±0.01^{c}	0.11±0.02^{a}	2554	2554	vanilla
	Aldehydes	18.33±1.61	20.16±1.21	23.96±2.47	25.06±3.94	13.33±0.45	18.03±1.83	21.39±4.97	21.44±4.78			

17	6-Methyltridecane	ND	ND	ND	0.42±0.26	ND	ND	ND	0.99±0.06	1300	1300	
18	3-Methyltridecane	0.69±0.25^{a}	0.35±0.14^{bc}	0.53±0.22^{ab}	0.13±0.00^{c}	0.79±0.25^{a}	0.41±0.07^{ab}	0.59±0.19^{ab}	0.24±0.04^{b}	1368	1368	
19	Tetradecane	1.36±0.88^{a}	1.14±0.68^{a}	1.23±0.77^{a}	0.68±0.36^{a}	1.29±0.94^{a}	1.18±0.27^{a}	1.33±0.67^{a}	0.74±0.29^{a}	1400	1400	
20	Hexadecane	1.70±0.65^{a}	1.64±0.27^{a}	1.75±0.52^{a}	1.05±0.34^{a}	1.33±0.49^{ab}	1.68±0.34^{a}	1.88±0.36^{a}	0.62±0.10^{b}	1600	1600	alkane
	Alkanes	3.75±1.75	3.13±1.01	3.52±1.47	2.28±0.17	3.41±1.69	3.27±0.26	3.80±0.85	2.59±0.18			
21	Styrene	1.03±0.41^{a}	0.29±0.09^{b}	0.39±0.06^{b}	0.15±0.04^{b}	1.44±0.40^{a}	0.35±0.07^{b}	0.40±0.09^{b}	0.18±0.09^{b}	1251	1251	balsamic, gasoline
22	Naphthalene	0.88±0.12^{b}	0.88±0.05^{b}	1.05±0.06^{a}	0.55±0.07^{c}	0.92±0.07^{a}	0.97±0.16^{a}	0.98±0.06^{a}	0.54±0.09^{b}	1730	1730	tar
23	*trans*-Calamenene	0.13±0.02^{b}	0.13±0.02^{b}	0.11±0.07^{b}	0.15±0.01^{b}	0.09±0.01^{a}	0.12±0.04^{a}	0.09±0.03^{a}	0.11±0.02^{a}	1828	1828	herb, spice
24	2-Methylnaphthalene	0.47±0.14^{a}	0.16±0.14^{b}	0.32±0.20^{ab}	0.17±0.01^{b}	0.50±0.16^{a}	0.48±0.38^{a}	0.34±0.25^{a}	0.19±0.01^{a}	1877	1877	floral sweet floral woody
25	Fluorene	0.05±0.04^{a}	0.06±0.01^{a}	0.03±0.02^{a}	0.03±0.01^{a}	0.05±0.04^{a}	0.05±0.01^{a}	0.02±0.02^{a}	0.03±0.00^{a}	2324	2324	
26	Benzophenone	0.06±0.01^{a}	0.02±0.02^{b}	0.03±0.01^{b}	ND	0.05±0.00^{a}	0.02±0.01^{b}	0.02±0.02^{b}	ND	2469	2469	

27	Dibutyl phthalate	0.86±0.84[a]	0.27±0.12[a]	0.19±0.07[a]	0.14±0.02[a]	0.53±0.32[a]	0.30±0.28[a]	0.28±0.32[a]	0.09±0.02[a]	2675	2675	
	Aromatics	3.48±1.35	1.80±0.14	2.11±0.40	1.19±0.11	3.57±0.90	2.30±0.76	2.15±0.67	1.14±0.22			
28	Acetic acid, methyl ester	0.22±0.05[a]	0.02±0.03[b]	0.02±0.03[b]	0.03±0.03[b]	0.35±0.02[a]	ND	0.03±0.05[b]	ND	835	835	ethereal
29	Ethyl Acetate	1.06±0.15[a]	0.18±0.03[b]	0.14±0.06[b]	0.14±0.03[b]	2.01±0.23[a]	0.20±0.02[b]	0.23±0.06[b]	0.05±0.04[b]	888	888	pineapple
30	Methyl tetradecanoate	0.32±0.14[a]	0.04±0.07[b]	ND	0.32±0.11[a]	0.87±0.18[a]	0.16±0.06[b]	0.05±0.08[b]	0.55±0.15[a]	2000	2000	orris
31	(*Z*)-9-Octadecenoic acid, methyl ester	0.54±0.30[ab]	0.05±0.09[b]	ND	1.23±0.99[a]	0.87±0.63[a]	ND	ND	0.53±0.53[a]	2447	2447	mild fatty
	Esters	2.14±0.50	0.29±0.10	0.16±0.09	1.72±1.15	4.10±0.58	0.36±0.07	0.31±0.12	1.13±0.65			
32	2-Ethylfuran	ND	ND	ND	0.12±0.04	ND	ND	ND	0.08±0.02	946	946	chemical
33	2-Propylfuran	ND	ND	ND	0.07±0.06	ND	ND	ND	0.02±0.03	1029	1029	
34	2-n-Butylfuran	0.15±0.02[b]	0.07±0.06[b]	0.10±0.09[b]	0.73±0.20[a]	0.09±0.08[b]	ND	ND	0.51±0.1 [a]	1133	1133	spicy
35	2-Pentylfuran,	3.02±0.20[b]	2.30±0.06[b]	2.40±0.43[b]	16.10±4.68 [a]	2.16±0.14[b]	1.66±0.31[b]	1.87±0.53[b]	12.82±2.68 [a]	1231	1231	green bean, butter

36	2-n-Heptylfuran	ND	ND	ND	0.09±0.08	ND	ND	ND	0.05±0.09	1432	1432	green
	Furans	3.17±0.22	2.37±0.05	2.50±0.52	17.11±5.04	2.25±0.16	1.66±0.31	1.87±0.53	13.47±2.88			
37	2-Heptanone	0.28±0.02[b]	0.13±0.11[b]	0.20±0.04[b]	0.62±0.14[a]	0.33±0.21[ab]	0.11±0.09[b]	0.13±0.11[ab]	0.51±0.13[a]	1180	1180	soap
38	2-Octanone	0.69±0.12[b]	0.64±0.17[b]	1.34±0.27[a]	0.19±0.05[c]	0.80±0.25[b]	0.93±0.08[b]	1.85±0.33[a]	0.22±0.05[c]	1283	1283	soap, gasoline
39	6-Methyl-5-hepten-2-one	ND	ND	ND	0.17±0.04	ND	ND	ND	0.13±0.03	1336	1336	pepper, mushroom, rubber
40	2-Nonanone	ND	ND	ND	0.26±0.07	ND	ND	ND	0.25±0.05	1387	1387	hot milk, soap, green
41	3-Nonen-2-one	0.46±0.07[a]	0.33±0.08[a]	0.44±0.21[a]	0.46±0.13[a]	0.24±0.08[a]	0.16±0.15[a]	0.24±0.08[a]	0.21±0.05[a]	1509	1509	fruity berry fatty oily ketonic weedy spicy licorice
42	6-Undecanone	ND	ND	ND	0.54±0.07	ND	ND	ND	0.35±0.09	1526	1526	orange, fresh, green
43	Geranylaceton	1.11±0.21[a]	1.05±0.10[a]	1.22±0.19[a]	1.15±0.14[a]	0.88±0.10[a]	1.00±0.25[a]	1.02±0.05[a]	0.63±0.13[a]	1853	1853	magnolia, green

44	2-Pentadecanone	0.09±0.16^{a}	ND	0.08±0.14^{a}	0.19±0.02^{a}	ND	0.38±0.06^{a}	0.22±0.20^{ab}	0.21±0.04^{ab}	2009	2009	
	Ketones	2.63±0.47	2.15±0.23	3.28±0.68	3.59±0.64	2.25±0.62	2.58±0.34	3.46±0.33	2.50±0.53			
45	Methylene chloride	0.18±0.07	ND	ND	ND	0.15±0.03^{a}	0.01±0.01^{a}	ND	ND	925	925	
46	2-Pentylthiophene	ND	ND	ND	0.64±0.10	ND	ND	ND	0.16±0.28	1454	1454	sweet, fruit
47	Isophorone	ND	ND	ND	ND	0.29±0.50^{b}	0.58±0.50^{ab}	0.62±0.54^{ab}	1.44±0.36^{a}	1586	1586	
48	Cedrol	2.84±0.61^{a}	1.52±0.91^{ab}	1.34±1.08^{b}	0.18±0.16^{b}	2.81±0.69^{a}	1.23±0.89^{ab}	1.74±1.81^{ab}	0.16±0.14^{b}	2109	2109	
49	Indole	0.34±0.08^{b}	0.38±0.03^{ab}	0.37±0.03^{b}	1.08±0.74^{a}	2.40±0.44^{a}	2.13±0.44^{a}	2.17±0.34^{a}	4.11±2.09^{a}	2436	2436	mothball, burnt
	Others	3.36±0.63	1.90±0.88	1.71±1.09	1.90±0.92	5.65±0.97	3.94±0.29	4.52±1.03	5.87±2.53	1287	1287	

ND: not detected.

Means with the same superscript letter were not significantly different (p <0.05).

a: calculated retention index were determined using n-alkanes C6 to C24 as external standards on a DB-Wax column.

b: RI_{ref} were obtained from https://webbook.nist.gov/.

c: odor description were obtained from https://www.flavornet.org/ and http://www.thegoodscentscompany.com/.

RR: raw rice; WR: washed rice; PR: presoaked rice; CR: cooked rice; ND: not detected.
